# Sodium metavanadate induced cognitive decline, behavioral impairments, oxidative stress and down regulation of myelin basic protein in mice hippocampus: Ameliorative roles of β‐spinasterol, and stigmasterol

**DOI:** 10.1002/brb3.1014

**Published:** 2018-06-01

**Authors:** Olamide Elizabeth Adebiyi, James Olukayode Olopade, Funsho Olakitike Olayemi

**Affiliations:** ^1^ Department of Veterinary Physiology and Biochemistry University of Ibadan Ibadan Nigeria; ^2^ Department of Veterinary Anatomy University of Ibadan Ibadan Nigeria

**Keywords:** β‐spinasterol, antioxidant, behavior, stigmasterol, vanadium

## Abstract

**Introduction:**

Exposures to toxic levels of vanadium and soluble vanadium compounds cause behavioral impairments and neurodegeneration via free radical production. Consequently, natural antioxidant sources have been explored for effective and cheap remedy following toxicity. *Grewia carpinifolia* has been shown to improve behavioral impairments in vanadium‐induced neurotoxicity, however, the active compounds implicated remains unknown. Therefore, this study was conducted to investigate ameliorative effects of bioactive compounds from *G. carpinifolia* on memory and behavioral impairments in vanadium‐induced neurotoxicity.

**Methods:**

Sixty BALB/c mice were equally divided into five groups (A–E). A (control); administered distilled water, B (standard); administered α‐tocopherol (500 mg/kg) every 72 hr orally with daily dose of sodium metavanadate (3 mg/kg) intraperitoneally, test groups C, and D; received single oral dose of 100 μg β‐spinasterol or stigmasterol (bioactive compounds from *G. carpinifolia*), respectively, along with sodium metavanadate and the model group E, received sodium metavanadate only for seven consecutive days. Memory, locomotion and muscular strength were accessed using Morris water maze, Open field and hanging wire tests. In vivo antioxidant and neuroprotective activities were evaluated by measuring catalase, superoxide dismutase, MDA, H_2_O_2_, and myelin basic protein (MBP) expression in the hippocampus.

**Results:**

In Morris water maze, stigmasterol significantly (*p *≤* *0.05) decreased escape latency and increased swimming time in target quadrant (28.01 ± 0.02; 98.24 ± 17.38 s), respectively, better than α‐tocopherol (52.43 ± 13.25; 80.32 ± 15.21) and β‐spinasterol (42.09 ± 14.27; 70.91 ± 19.24) in sodium metavanadate‐induced memory loss (112.31 ± 9.35; 42.35 ± 11.05). β‐Spinasterol and stigmasterol significantly increased exploration and latency in open field and hanging wire tests respectively. Stigmasterol also increased activities of antioxidant enzymes, decreased oxidative stress markers and lipid peroxidation in mice hippocampal homogenates, and increased MBP expression.

**Conclusions:**

The findings of this study indicate a potential for stigmasterol, a bioactive compound from *G. carpinifolia* in improving cognitive decline, motor coordination, and ameliorating oxidative stress in vanadium‐induced neurotoxicity.

## 1 INTRODUCTION

Vanadium is a transition metal (Habib & Ibrahim, 2011), which has been recognized to be acutely toxic by most routes of introduction following environmental exposure in large doses. It is a preferred metal for the production of special steels and temperature‐resistant alloys because it is one of the lightest high‐strength metals (Bunting, 2006). Predominantly, vanadium compounds are often released into the environment in large quantities by burning fossil fuels. Environmental exposures to vanadium (V) produce several adverse health effects in animals and humans with its accumulation in the soil, groundwater, and plants that may be consumed by them (Pyrzynska & Weirzbicki, 2004).

Humans and animals are potentially exposed to a range of vanadium compounds, commonly, sodium orthovanadate, vanadium pentoxide, sodium metavanadate, vanadyl sulfate, and ammonium metavanadate (Farid, Abozid, & Mahmoud, 2012). Previous studies have shown that exposure of vanadium in its different forms may cause central nervous system (CNS) depression, tremor, behavioral deficits, neurasthenia, and other severe motor deficits including vegetative symptoms (WHO, 2000). Another study also showed that inhaled vanadium pentoxide damage the nigrostriatal dopaminergic system in rodent models (Avila‐Costa et al., 2004); V‐induced neurotoxicity has been closely linked to the persuasion of oxidative stress that leads to reactive oxygen species (ROS) generation and lipid peroxidation. V generally accumulates in the brain (Garcia, Biancardi, & Quiroga, 2005). However, living cells possess diverse mechanisms to maintain metal concentrations at levels that do not exceed cellular requirements. For example, chelation by either glutathione (GSH) is found in all organisms participating in multiple metabolic processes, such as intracellular redox state regulation, inactivation of ROS, transport of GSH‐conjugated amino acids, and other molecules (Jozefczak, Remans, Vangronsveld, & Cuypers, 2012).

Despite the extensive uses of vanadium and its attendant health effects, in particular in the CNS, compounds to mitigate these effects are not well‐characterized. The intrinsic limitations and variability in the efficacy of previous heavy metal chelating agents has necessitated the need for the development of novel therapeutic agents with various modes of actions, especially from medicinal plants (because of their perceived reduced side effects as compared to synthetic drugs).

Our previous studies have shown that ethanol crude extract of *Grewia carpinifolia* leaf possesses to an extent CNS and antioxidant activities (Adebiyi, Olayemi, Ning‐Hua, & Guang‐Zhi, 2017; Adebiyi, Olayemi, & Olopade, 2016; Adebiyi, Olopade, Olopade, & Olayemi, 2016). Consequently, since antioxidant and chelating agents have been proposed for the treatment of vanadium poisoning, the present study was designed to isolate bioactive compounds from *G. carpinifolia* as well as investigate their possible beneficial effects against brain injury induced by vanadium.

## 2 MATERIALS AND METHODS

### 2.1 Experimental animals

Sixty male BALB/c mice of about 4 weeks old with average weight of 18–21 g were obtained and housed at the Departmental Animal House holding unit. The animals were housed under standard conditions of temperature (25 ± 2°C) and light (approximately 12/12 hr light–dark cycle), fed on standard diet, fresh water ad libitum and acclimatized to laboratory conditions 2 weeks before the commencement of the experiment. All animal handling, care, and treatment was carried out in strict accordance with the OECD approved Standard Operation Procedures in the use of animals and specifically reviewed and approved by the Animal Care and Use Research Ethics Committee of the University (UI‐ACUREC/App/2016/025). Efforts were made to minimize pain, suffering, and number of animal used.

The mice were randomly divided into five groups of twelve animals per group. Group A received distilled water throughout the experimental period and served as control, group B; the standard group received vitamin E (500 mg/kg) every 72 hr orally along with a daily dose of sodium metavanadate at 3 mg/kg (Mustapha et al.*,* 2014) intraperitoneally (i/p) for 7 days consecutively, the test groups C, and D received a single oral dose of 100 μg β‐spinasterol and stigmasterol, respectively, along with sodium metavanadate at 3 mg/kg i/p for 7 days consecutively and the model group E received sodium metavanadate only at 3 mg/kg i/p for 7 days consecutively.

### 2.2 β‐Spinasterol and stigmasterol

Bioactive compounds (β‐spinasterol and stigmasterol) isolated from *G. carpinifolia* with potent antioxidant activity in various in vitro models (Table 1) were used for the present study.

**Table 1:** IC_50_value of ABTS and DPPH radicals scavenging activity of spinasterol and stigmasterol

	IC_50_ (mg/ml) of ABTS	IC_50_ (mg/ml) of DPPH
Spinasterol	0.38	0.70
Stigmasterol	0.40	0.61
Ascorbic acid (reference standard)	0.21	0.98

*Note*

IC_50_: inhibitory concentration 50%; ABTS: 2,2′‐azinobis‐3‐ethylbenzothiozoline‐6‐sulfonic acid radical; DPPH: 1,1‐diphenyl‐2‐picryl hydroxyl.

### 2.3 Chemicals

Sodium metavanadate (NaO_3_V) (Sigma, St. Louis, MO, USA), myelin basic protein (MBP) antibody (Abcam, Cambridge, UK), nitric acid (Fisher Scientific, Pittsburgh, PA, USA), anti‐mouse MBP antibody (Abcam), ethanol and the rest of the solvents used were of analytical grade.

### 2.4 Behavioral tests

#### 2.4.1 Morris water maze

The Morris water maze is a circular pool (110 cm in diameter) with water (25 ± 1°C), 30 cm in height) with a hidden circular escape platform (12 cm in diameter) as described by D’Hooge and De Deyn (2001). The pool was marked North, South, East, and West and the hidden platform was placed in a particular spot. Each mouse was dropped into the pool and expected to find the platform, and the length of time it takes to find the platform was recorded. On the fourth day, a single probe trial was given to test the mouse’s spatial memory in the water maze while the platform was removed (Akbari, Naghdi, & Motamedi, 2006). The time it spent in the target quadrant and the number of times it crossed the zone where the platform was initially located was recorded manually with a stopwatch (Golchin, Vahidi, & Shabani, 2013).

#### 2.4.2 Open field test

Each mouse was placed in the center of a square cage (120 cm × 120 cm) drawn with red ink. The floor was divided into 20 cm squares drawn with black ink. The mouse was allowed to move freely around the open field and explore the environment for 5 min. Frequency of line crossing, rearing, stretch‐attend posture, number of fecal boli, center square, and freezing durations were scored as described by Brown, Corey, and Moore (1999).

#### 2.4.3 Hanging‐wire test

This test was performed as described by Van Putten et al. (2012), concisely, the forepaws of each mouse were placed on a horizontally suspended wire (1 mm in diameter), placed 47 cm above a soft foam landing area. The mouse was then timed from the moment it was placed on the wire until it dropped from the wire.

### 2.5 In vivo antioxidant assay

At the end of study period, mice were sacrificed by cervical dislocation. Hippocampus was isolated and prepared as hippocampal homogenate. Malondialdehyde (MDA), H_2_O_2_ levels and the activities of superoxide dismutase, catalase (CAT) in were determined. MDA level was estimated by determining the accumulation of thiobarbituric acid reactive substances (TBARS) in the hippocampal homogenate as described by Mihara and Uchiyama (1978). The levels of H_2_O_2_ was determined as described by Fossati, Prencipe, and Berti (1980), activities of SOD and CAT were determined by recording the ability to inhibit cytochrome C and the rate of decrease in H_2_O_2_ respectively (Thukhum‐mee, Wattanathorn, Muchimapura, & Bunchonglikitkul, 2012).

### 2.6 Immunohistochemistry

The mice were anesthetized with ketamine (intraperitoneal, Parke‐Davis Ltd, India) and perfused with 4% paraformaldehyde (PFA) in PBS. The brain was dissected, processed, and embedded in paraffin blocks (Ramos‐Vara, 2011). Briefly, brain sections were immersed in 4% phosphate buffer formalin. The sections were deparaffinized and dehydrated with gradient xylene and alcohol and antigen was retrieved in Na citrate buffer. Antigen retrieval was done by microwave heating in 10 mM citrate buffer for 25 min, with subsequent peroxidase quenching in 3% H_2_O_2_/methanol. All the sections were blocked in 2% skimmed milk and probed with anti‐MBP rat monoclonal antibody (1:1,000; Abcam, SMI‐94, mouse, #GR207556‐1, RRID: http://scicrunch.org/resolver/AB_991778) for 16 hr at 4°C. Detection of bound antibody was done using HRP‐conjugated secondary antibody (Abcam, AB6819, donkey anti‐mouse, #GR866‐75‐5, RRID: http://scicrunch.org/resolver/AB_954883). Incubation was done in Avidin‐biotin complex (ABC) (Otani et al., 1999) solution and the reaction product was enhanced with diaminobenzidine (VectorStain, #SK‐4100) chromogen for 6–10 min, with subsequent dehydration in ethanol and mounting on salinized slides before visualizing under the microscope.

The expression of MBP was quantified using the ImageJ software called Fiji (Schindelin et al., 2012).

### 2.7 Statistical analysis

Data are presented as mean ± SEM. Data were analysed by two‐way analysis of variance (ANOVA) for repeated exposures and subsequently Bonferroni post‐test using Graph pad Prism version 5 (Windows^®^ Graphpad software) for mean comparisons. *p*‐Values ≤0.05 were considered statistically significant.

## 3 RESULTS

### 3.1 Morris Water Maze

The Morris Water Maze Task is a behavioral task that has been developed to study both learning and memory in animals (Sutherland & Rudy, 1989). In addition, the probe trail analyses reference memory and is determined by preference for the platform area when the platform is absent (D’Hooge & De Deyn, 2001).

**Figure 1:**
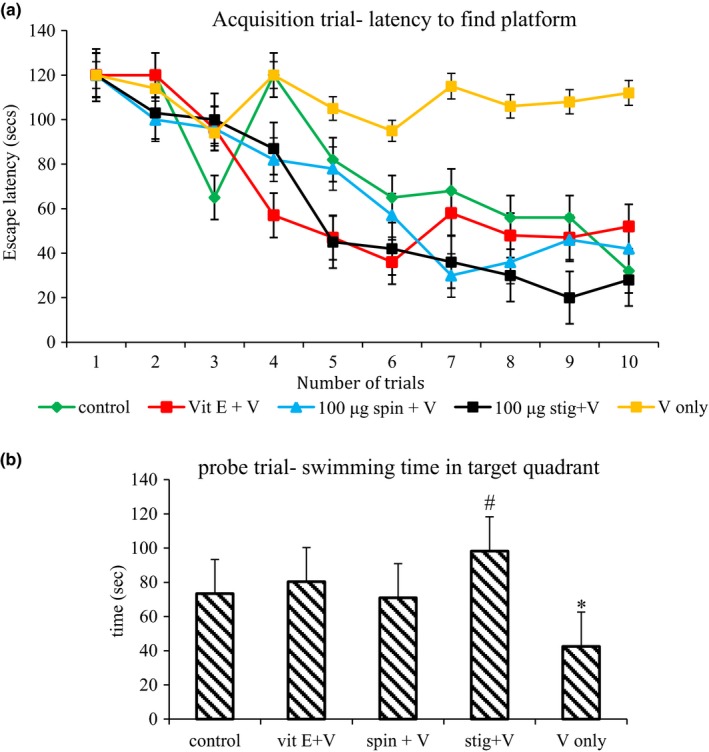
Effect of concurrent administration of β‐spinasterol or stigmasterol on learning and memory in mice following acute vanadium toxicity. *n* = 12; Vit E: α‐tocopherol; V: sodium metavanadate; spin: β‐spinasterol; stig: stigmasterol. *Statistically different from the normal control at *p *≤* *0.05, ^#^ *p *≤* *0.05 versus sodium metavanadate untreated group

Acquisition Trails‐ Two‐way ANOVA (day × group) for repeated measures showed that each of the groups had a significant effect on escape latency. Similarly a significant day effect on escape latency was observed, which indicated that mice improved over the course of training trails in all groups except the group treated with sodium metavanadate only. Successive comparisons additionally suggested that no difference was observed between the control and test groups on escape latency. The mice injected with sodium metavanadate took a longer time to find the platform than did mice in the test groups (Figure 1a) but the escape latency became significantly (*p *=* *0.028) longer on days 2 and 3. Stigmasterol significantly reduced escape latency (E) (day 1, E = 100 s; day 3, E = 15 s) in comparison to the standard control group of α‐tocopherol (day 1, E = 114 s; day 3, E = 33 s). The groups treated with β‐spinasterol also had a significant (*p *=* *0.034) decrease in the escape latency over time.

In the probe trial, the time spent in the target quadrant was significantly (*p *=* *0.042) lower in the model group treated with only sodium metavanadate. β‐Spinasterol slightly increased the swimming time in the target quadrant but without any significant (*p *=* *0.089) difference. By divergence, the swimming time in the target quadrant was markedly (*p *=* *0.001) increased by stigmasterol when compared to all other groups (Figure 1b).

### 3.2 Open field test

**Figure 2:**
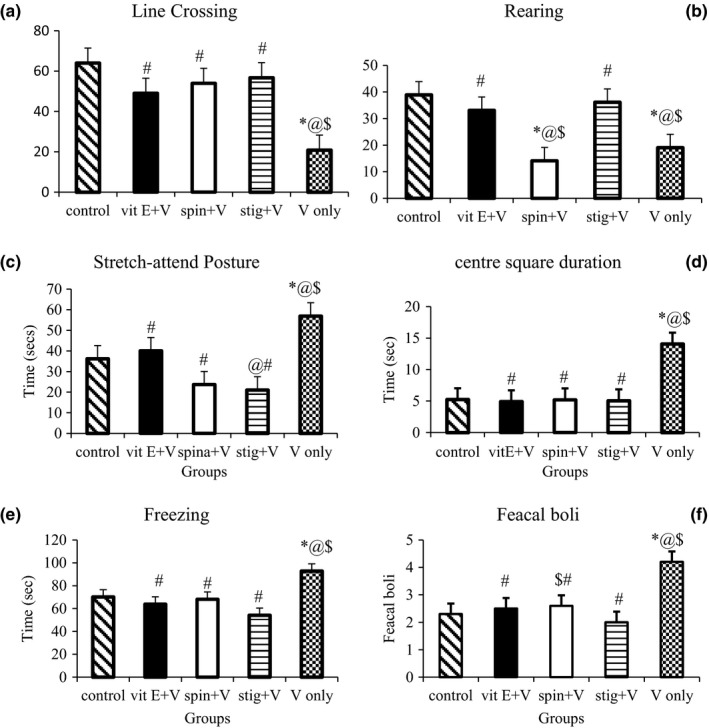
Number of line crossings and rearings, stretch‐attend posture, centre square duration, freezing, and fecal boli in the open field following concurrent administration of β‐spinasterol or stigmasterol and vanadium. *n *=* *12, Vit E: α‐tocopherol; V: sodium metavanadate; spin: β‐spinasterol; stig: stigmasterol ^*^significantly (*p *≤* *0.05) different from the control; ^@^significantly (*p *≤* *0.05) different from the α‐tocopherol + sodium metavanadate group, ^$^significantly (*p *≤* *0.05) different from the stigmasterol + sodium metavanadate group; ^**#**^significantly different from the sodium metavanadate group

The Open‐field test evaluates locomotor activities and anxiety behavior in rodents (Stanford, 2007). The frequency of line crossings and rearing are measures of locomotor and explorative activity respectively. Stretch‐attend posture (SAP) is a “risk‐assessment” behavior which indicates that the animal is hesitant to move from its present location to a new position (Blanchard, McKittrick, & Blanchard, 2001). A high frequency of SAP and fecal boli indicates a higher level of anxiety. The number of line crossed by mice in the control and test groups; treated with β‐spinasterol and stigmasterol was not significantly (*p *=* *0.105) different, however line crossing was significantly (*p *=* *0.042) lower in group administered with sodium metavanadate only (Figure 2a). The rearing frequency of experimental mice was higher in the test groups when compared with the model group treated with sodium metavanadate only (Figure 2b). Mean duration of stretch‐attend postures of animals were 36.31 ± 5.95, 40.09 ± 6.75, 23.64 ± 6.34, 21.70 ± 2.59 and 56.91 ± 7.02 s for control, groups B (administered with α‐tocopherol), C (β‐spinasterol), D (stigmasterol), and E (sodium metavanadate only) respectively (Figure 2c). The center square duration of the test groups was not significantly different from those of the control and standard group administered with α‐tocopherol, conversely a significant increase in time spent at the center of the box was recorded in the sodium metavanadate only group when compared with the other groups (Figure 2d). The freezing duration and number of fecal boli were similar across the groups except in the sodium metavanadate only group where significantly higher values were observed (Figure 2e and f).

### 3.3 Hanging wire test

**Figure 3:**
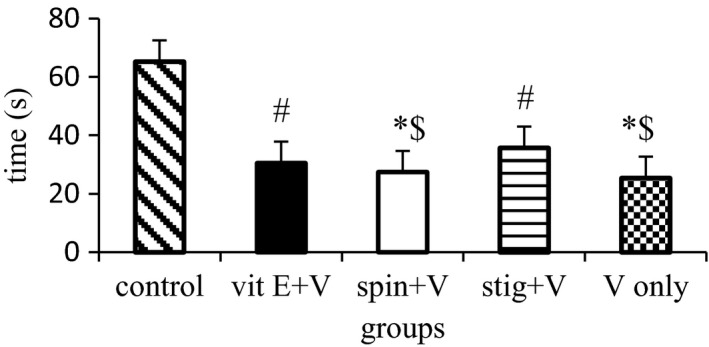
Hanging latency following concurrent administration of β‐spinasterol or stigmasterol and vanadium. *n *=* *12, Vit E: α‐tocopherol; V: sodium metavanadate; spin: β‐spinasterol; stig: stigmasterol *statistically different from the control at *p *≤* *0.05

The hanging wire test is performed in order to demonstrate motor neuromuscular impairment and motor coordination in mice (Crestani et al., 2001). Hanging latency was significantly (*p *=* *0.026) decreased in the sodium metavanadate only group when compared with control mice. Conversely, coadministration with stigmasterol improved significantly (*p *=* *0.024) the grip strength and hanging latency when compared to the standard group. The hanging latency in the β‐spinasterol treated group was however similar with that of the model group treated with sodium metavanadate only (Figure 3).

### 3.4 In vivo antioxidant study

**Figure 4:**
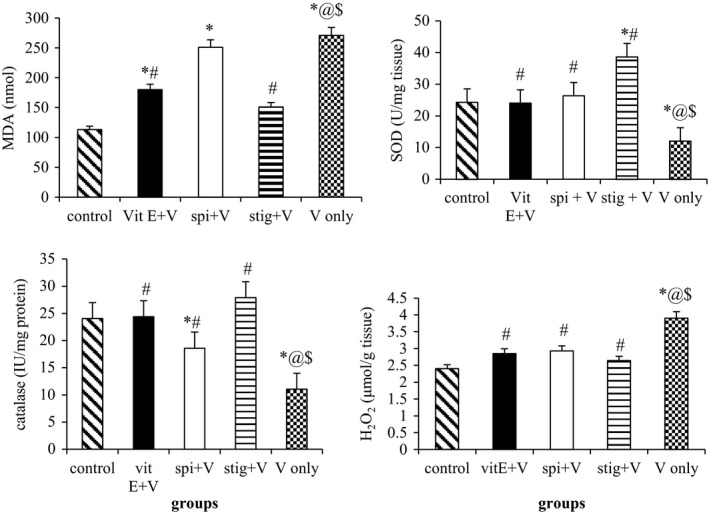
Effects of α‐tocopherol, β‐spinasterol or stigmasterol on vanadium‐induced oxidative stress in mice. *n *=* *5; Vit E: α‐tocopherol; V: sodium metavanadate; spin: β‐spinasterol; stig: stigmasterol; ^*^significantly (*p *≤* *0.05) different from the control; ^@^significantly (*p *≤* *0.05) different from the α‐tocopherol + sodium metavanadate group; ^$^significantly (*p *≤* *0.05) different from the stigmasterol + sodium metavanadate group; ^#^significantly different from the sodium metavanadate group

Daily administration of sodium metavanadate for 7 days in mice resulted in a significant (*p *=* *0.032) reduction in the activities of SOD, catalase (CAT), and increased hippocampal MDA and H_2_O_2_ levels (Figure 4). However, concomitant administration of stigmasterol decreased elevated hippocampal MDA and restored altered activities of these enzymes to values comparable to control group. The catalase activities in groups coadministered with β‐spinasterol and sodium metavanadate were significantly (*p *=* *0.019) lower than the other groups.

### 3.5 Immunohistochemistry

**Figure 5:**
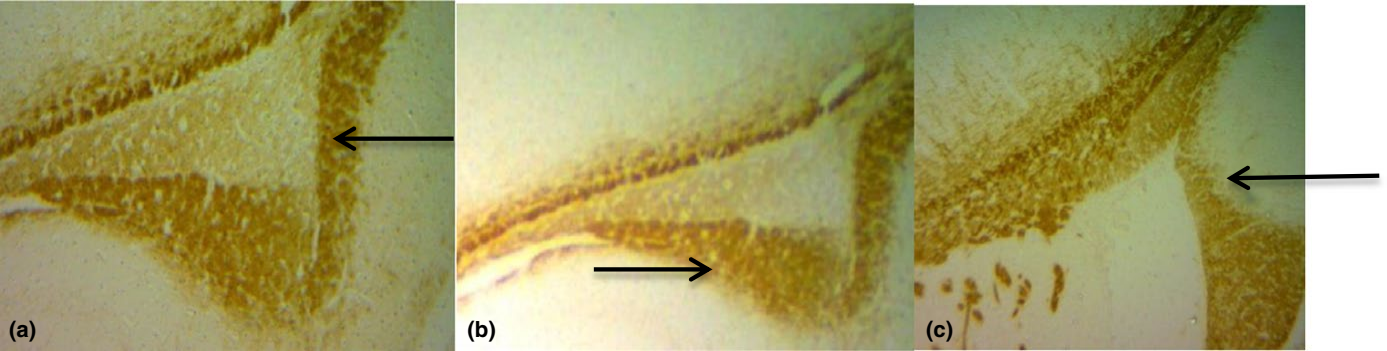
MBP immunolabeled myelin fibers (arrows) in the hippocampus ×100 (a) control group showed that myelin fibers were arranged closely and uncluttered (b) coadministration with stigmasterol and sodium metavanadate showing closely and orderly arranged myelin fibers (c) administration with sodium metavanadate showing poor staining and discontinuity of myelin fibers in the dendate gyrus. MBP, myelin basic protein

**Figure 6:**
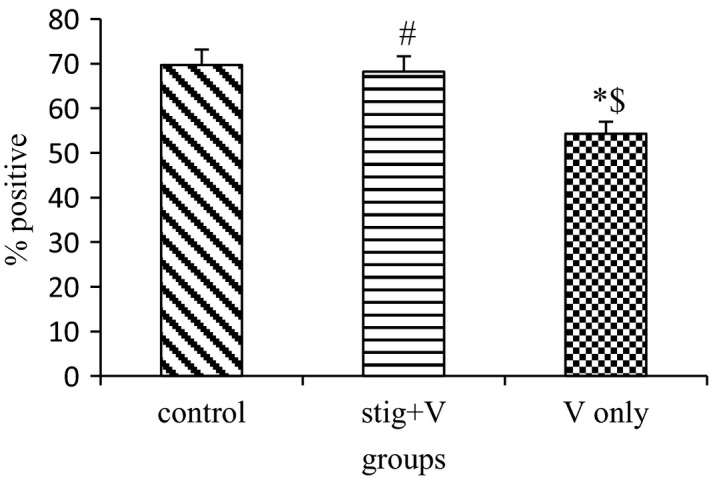
Quantification of myelin basic protein expression using ImageJ^®^ software. *n *=* *5; V: sodium metavanadate; stig: stigmasterol; ^*^significantly (*p ≤ *0.05) different from the control; ^@^significantly (*p *≤* *0.05) different from the α‐tocopherol + sodium metavanadate group; ^$^significantly (*p *≤* *0.05) different from the stigmasterol + sodium metavanadate group; ^#^significantly different from the sodium metavanadate group

Myelin basic protein immunohistochemical staining showed a down regulation of MBP in vanadium exposed group compared with the pure compounds treated groups and control in the hippocampus (Figure 5). The discontinuity of myelin observed in the group administered with sodium metavanadate only (Figure 5c) was restored by concurrent administration of stigmasterol (Figure 5b). The number of cells expressing the myelin basic protein was thereafter quantified with the ImageJ^®^ software (Figure 6). The expression of MBP in the hippocampus was significantly higher in the group coadministered with stigmasterol when compared with the model group of sodium metavanadate only.

## 4 DISCUSSION

We evaluated the effect of bioactive compounds (β‐spinasterol and stigmasterol) from ethanol extract *G. carpinifolia* leaf extract on vanadium‐induced learning and memory, motor coordination, and behavioral deficits using the Morris water maze, open field and hanging wire tests respectively. In the present study, the learning curve of the acquisition trials in the Morris Water Maze revealed that sodium metavanadate caused a deficit in learning; this is an indication of neurodegeneration in hippocampus (Cassel, Mathis, Majchrzak, Moreau, & Dalrymple‐Alford, 2008; Devi, Diwakar, Raju, & Kutty, 2003). The ability of β‐spinasterol and stigmasterol to decrease the time to find the platform with subsequent trials indicates that these bioactive compounds increase learning of spatial location of the escape platform. In the probe trail of the present study, we found that, administration of sodium metavanadate alone showed significant memory impairments. This is in consonance with reports by Folarin, Olopade, Onwuka, and Olopade (2016) that vanadium exposure led to loss of memory acumen but in contrast to their finding of no variation in learning abilities, the variance in these results may be ascribed to the difference in duration of exposure. Stigmasterol significantly shortened the escape latency prolonged by sodium metavanadate injection after 2 days of training. Interestingly, using the escape latency as an index of learning and memory, this compound showed stronger improving ability compared to that of α‐tocopherol and β‐spinasterol. In addition, stigmasterol also increased the swimming time in the quadrant where the platform was previously placed. This indicates that stigmasterol improved spatial learning and memory in mice following vanadium‐induced neurotoxicity.

In the present study, stigmasterol from *G. carpinifolia* extract significantly increased exploratory activity as evidenced by the numbers of line crossed, rearing and reduction in center square duration. Likewise in this study, mice exposed to vanadium, unlike the test and control groups, recorded decreases in locomotor activity and exploration with a concomitant increase in anxiety levels in the open field test. This agrees with similar results obtained by Domingo (1996) and Soazo and Garcia (2007). Olopade, Fatola, and Olopade (2011) attributed this reduced exploration to vanadium‐induced muscular weakness. Stigmasterol administration probably led to increase alertness subsequent to increased locomotor activity, indicating its anxiolytic property as supported by the correlation between anxiety and locomotion reported by (Bernatova, Puzserova, Sestakova, & Mach, 2011). In several studies compounds from plants have been shown to have anxiolytic activity (Gadekar, Sourabh, & Jitender, 2011; Herrera‐Ruiz, Román‐Ramos, Zamilpa, Tortoriello, & Jiménez‐Ferrer, 2008; Sandeep & Suresh, 2010). Their anxiolytic effect has been attributed to its effect on gamma‐amino‐butyric‐acid (GABA), receptors in the CNS (Apu, Hossain, Rizwan, Bhuyan, & Jamaluddin, 2013) which may be the mechanism through which stigmasterol acts. This study provides experimental/scientific support for the folklore use of *G. carpinifolia* in the management of anxiety.

The ability of β‐spinasterol and stigmasterol to significantly increase latency in the hanging wire test might show their contribution to muscular strength in animal models. The significant increase in hanging latency and exploratory activity by stigmasterol in the hanging wire and open‐field, respectively, directly correlate with increased swimming time to reach the target platform in the acquisition trial in the Morris water maze suggesting that muscle activity and motor coordination improved by this compound.

### 4.1 In vivo antioxidant study

The increase in oxidative stress and overwhelming of in vivo antioxidant defense system following vanadium induced toxicity has not been disputed (Sasi, Haider, El‐Fakhri, & Gwarsha, 1994; Saxena, Arya, Saxena, & Shukla, 2013). SOD is the antioxidant enzyme responsible for the dismutation of the O^2^‐ generated during vanadium metabolism (Ibrahim, Froberg, Wolf, & Rusyniak, 2006) to H_2_O_2_ which is then converted into water by glutathione and catalase (Abreu & Cabelli, 2010). The observed decrease in both SOD and catalase activities in sodium metavanadate‐treated mice in this study strongly suggest an overwhelming superoxide radical generation leading to mopping up (use‐up) of the enzymes and H_2_O_2_ formation following vanadium administration. The in vivo antioxidant potential of both β‐spinasterol and stigmasterol after cotreatment with sodium metavanadate is evidenced by a significant improvement in the activities of SOD and CAT. This can be linked to the ability of these compounds in biological system to increase the activities of antioxidant enzymes so as to tackle the increased oxidative stress.

The significant increase in MDA and H_2_O_2_ levels in the sodium metavanadate group could be due to the presence of transition metal like Fe^2+^ in the brain, causing generation of highly reactive hydroxyl radicals (OH^−^) from the H_2_O_2_ through Fenton reaction as reported by Bhattacharya (2015). These ROS react with cellular macromolecules with consequent lipid peroxidation and depletion of sulfhydryl‐containing peptides leading to increase in MDA levels. These results strongly support the hypothesis that increased oxidative stress associated with an impaired antioxidant defense status is one of the mechanisms of vanadium toxicity (Ngwa et al., 2009). Our finding is also in congruence to that of Um et al. *(*2006) that relates abnormal alteration in MDA level to memory impairment.

Attenuation of profound reductions in the activities of CAT and SOD as well as reduction in hippocampal levels of MDA and H_2_O_2_ strongly indicate the possible antioxidant and anti‐lipoperoxidative effects of stigmasterol. In a previous study, we reported that crude ethanol extract of *G. carpinifolia* leaf improved spatial memory in rat through a mechanism that involved anti‐oxidative and neuro‐protective activities (Adebiyi, Olopade, et al., 2016). The crude extract was also able to reduce iron Fe^3+^ to Fe^2+^ and scavenged 2,4‐dinitrophenyl‐1‐picryl hydrazyl (DPPH) and 2,2′‐azinobis‐3‐ethylbenzothiozoline‐6‐sulfonic acid (ABTS) radicals in vitro (Adebiyi et al., 2017) via their reducing properties, they also donate hydrogen atom which breaks the chain of ROS. The present findings are in line with previous findings that the neuroprotective effects of various phytochemicals are associated with reduced levels of oxidative stress (Ikeda, Negishi, & Yamori, 2003; Joseph, Bartus, & Clody, 2005).

### 4.2 Immunohistochemistry

Myelin basic protein, is a basic membrane proteins synthesized by oligodendrocytes in the central nervous system (Boggs, 2006) and it is specific to nervous tissues (DeBruin et al., 2005). Myelin sheath is required for proper functioning of most long‐range axonal projections involved in motor or sensory functions of the brain (Barres, 2008) and nerve fiber with myelin confers faster impulse conduction (Baumann & Pham‐Dinh, 2001). Toxicants such as vanadium have been reported to cause oligodendrocyte death leading to myelin disruption before, during and after formation (Herring & Konradi, 2011; Todorich et al., 2011). In line with previous study, our findings showed that vanadium‐induced neurotoxicity, reduced expression of MBP significantly with pale areas and discontinuity of myelin fibers. Thus, the altered myelination of hippocampal axons may be responsible for the impaired learning and memory in the sodium metavanadate only group. Conversely, administration of stigmasterol significantly increased MBP expression. This is further supported by earlier findings which have stated that increased expression of MBP plays a role in the protection of brain (Wang, Tu, Huang, & Ho, 2012) suggesting that stigmasterol, a bioactive compound obtained from *G. carpinifolia* plays an ameliorative role following vanadium‐induced demyelination. This is also buttressed by previous findings that stigmasterol aids repair of damaged neurons by neuronal synthesis, restoration of synaptic activity and ultimately improve nerve impulse transmission (Park et al., 2012).

Therefore, considering that memory impairment and hippocampal pathology are prominent in several neurodegenerative conditions such as multiple sclerosis (Zhang, Liu, Fox, & Xiong, 2013) leading to spectrum of cognitive deficits and several behavioral impairment, the findings from this study have further led credence to the link between vanadium toxicity and neurodegenerative disorders and stigmasterol may be of therapeutic benefits.

## 5 CONCLUSION

The potentials of stigmasterol and β‐spinasterol which are pure bioactive compounds obtained from *G. carpinifolia* extract in improving cognitive decline, motor coordination and ameliorating oxidative stress in vanadium‐induced toxicity in mice model has been indicated. Furthermore, it can be concluded that stigmasterol protected against vanadium‐induced neurodegeneration better than α‐tocopherol and β‐spinasterol attributed to its improved cognitive, antioxidant, and myeloprotective activities. The activity of this compound should be further explored in amnesia, multiple sclerosis, and other degenerative conditions in which altered neurotransmission is known to play vital role in their pathogenesis.

## CONFLICT OF INTEREST

None declared.
